# Supplementary figures and images for: Enhanced industrial text classification via hyper variational graph-guided global context integration
Source: PeerJ Comput Sci. 2024 Jan 5;10:e1788. doi: 10.7717/peerj-cs.1788 (PMC10773843; doi:10.7717/peerj-cs.1788)

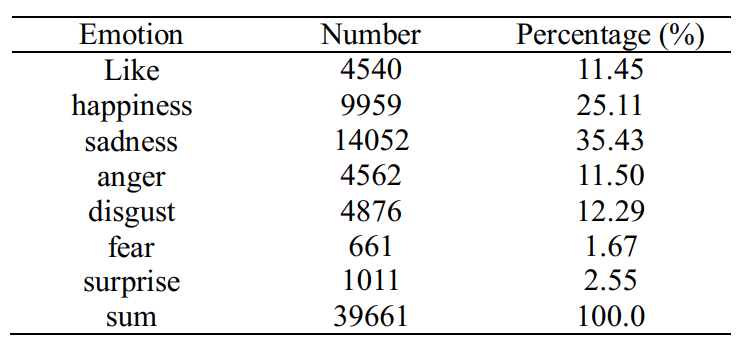

Supplement: Supplemental Information 2 — CLUEEmotion2020 dataset is emotion analysis corpus labeled with each sample annotated with one emotion label, and contains train, validation and test dataset. CHIP-CTC dataset is categorized based on the screening criteria utilized in clinical trials. It is sourced entirely from authentic clinical trial cases with the abbreviated data, and obtained from the standardized module within the public Chinese clinical website. N15News dataset is generated from New York Times with 24 categories and contains both text and image information in each news. Here we use the text with body tag for news classification as test dataset. PatentsDataset corpus comes from industrial domain texts extracted from China’s national patent database, with over 3500 self-built industrial equipment patent information used for industrial field text classification. [file peerj-cs-10-1788-s002.zip › data/CLUEEmotion2020/label_distribution.png]
